# Polymorphisms of selected Xenobiotic Genes contribute to the development of Papillary Thyroid Cancer susceptibility in Middle Eastern population

**DOI:** 10.1186/1471-2350-9-61

**Published:** 2008-07-05

**Authors:** Abdul K Siraj, Muna Ibrahim, Maha Al-Rasheed, Jehad Abubaker, Rong Bu, Shakaib U Siddiqui, Fouad Al-Dayel, Osama Al-Sanea, Abdulrahman Al-Nuaim, Shahab Uddin, Khawla Al-Kuraya

**Affiliations:** 1 Cancer Genomics, Research Centre, King Fahad National Center for Children's Cancer & Research, King Faisal Specialist Hospital and Research Centre, Riyadh, Saudi Arabia; 2 Department of Pathology and Laboratory Medicine, King Faisal Specialist Hospital and Research Centre, Riyadh, Saudi Arabia; 3 Health Sciences Centre, Saad Specialist Hospital, Al-Khobar, Saudi Arabia; 4 Department of Medicine, King Faisal Specialist Hospital and Research Centre, Riyadh, Saudi Arabia

## Abstract

**Background:**

The xenobiotic enzyme system that enables us to detoxify carcinogens exhibits identifiable genetic polymorphisms that are highly race specific. We hypothesized that polymorphisms of these genes may be associated with risk of thyroid cancer. To evaluate the role of genetic polymorphisms of xenobiotic genes in thyroid cancer, we conducted a hospital-based case-control study in Saudi population.

**Methods:**

223 incident papillary thyroid cancer cases and 513 controls recruited from Saudi Arabian population were analyzed for the association between polymorphisms in genes encoding folic acid metabolizing enzymes MTHFR and six xenobiotics-metabolizing enzymes including *CYP1A1 T3801C, C4887A, GSTP1 A1578G, C2293T, GSTM1, GSTT1*, NAT2 G590A, NQO*1 C609T, using PCR-RELP.

**Results:**

Among selected genes, *CYP1A1 C4887A *genotypes CA, AA and variant allele A demonstrated significant differences and greater risk of developing thyroid cancer comparing to wild type genotype CC (CA vs. CC; p < 0.0001, OR = 1.91, 95% CI = 1.36–2.70, AA vs. CC; p < 0.001, OR = 3.48, 95% CI = 1.74–6.96 and CA+AA vs. CC; p < 0.0001, OR = 2.07, 95% CI = 1.49–2.88). *GSTT1 *null showed 3.48 times higher risk of developing thyroid cancer (p < 0.0001, 95% CI = 2.48–4.88) while *GSTM1 *null showed protective effect (p < 0.05, OR = 0.72, 95% CI = 0.52–0.99). Remaining loci demonstrated no significance with risk.

**Conclusion:**

Of the 9 polymorphisms screened, we identified *GST, GSTM1 and CYP1A1 C4887A*, *m*ay be of importance to disease process and may be associated with papillary thyroid cancer risk in Saudi Arabian population.

## Background

Thyroid cancer is the most common endocrine malignancy, accounting for 3% of all cancers and is the eighth most common malignancy in the United States [1]. In contrast, in Kuwait, thyroid cancer ranks second, comprising 8% of all female cancers [2] and similar findings have been reported for other countries in the Gulf region [3]. Thyroid cancer is the fourth most common cancer and overall accounting for 6.1% in Saudi Arabia representing 3.8% male and 10.8% female malignant neoplasms, second to breast cancer [4]. Papillary thyroid carcinoma is the predominant type, accounting for almost all (approximately 90%) thyroid malignancies [5]. Although the exact etiology of thyroid cancer remains unknown, exposure to ionizing radiation is the only verified cause of thyroid carcinogenesis in humans, especially when exposure occurs at young age, although dietary iodine deficiency has also been linked to this pathology [6-8]. However, individuals without previous exposure to ionizing radiation can also develop thyroid cancers, usually defined as sporadic tumors [9], suggesting that other risk factors could also be involved in the etiology of sporadic tumors.

It has also been suggested that individuals possessing a modified ability to metabolize carcinogens are at increased risk of cancer [10-12]. For this reason, five (5) families of genes encoding for enzymes controlling oxidative stress have attracted our interest CYPs *(Phase I Cytochromes P450)*, GSTs *(Phase II Glutathione-S-Transferase)*, NADPH *(quinone oxidoreductase NQO1)*, NAT (*N*-acetyltransferases), and gene encoding folic acid metabolizing enzyme, Methylenetetrahydrofolate reductase (*MTHFR*).

*MTHFR *balances the pool of folate coenzymes in one carbon metabolism of DNA synthesis and methylation, both are implicated in carcinogenesis of many types of cancer. Previous reports concerning *MTHFR *gene polymorphisms suggests that individuals carrying the less active form of folate metabolizing enzyme will be at greater risk of cancer [10-12].

The *N*-acetyltransferases (NAT) are involved in the metabolism of drugs and environmental toxins. They catalyze the acetyltransfer from acetylcoenzyme A to an aromatic amine, heterocyclic amine or hydrazine compound. Sequence variations in the human *NAT1 *and *NAT2 *result in the production of NAT proteins with variable enzyme activity or stability, leading to slow or rapid acetylation. Therefore, genetic polymorphisms in *NAT1 *and *NAT2 *have been associated with drug-induced toxicities and disease [13-15].

NQO1 is acytosolic enzyme catalyzing a 2 electron reduction of quinone and preventing their participation in redox cycling and thus inoxidative stress [16]. Polymorphism within *NQO1 *has shown to be associated with increased risk of myeloid leukemia [17], bladder carcinoma [18].

CYP1A1 catalyzes the oxidation of *polycyclin aromatic hydrocarbons *(PAHs) to epoxides and is inducible by PAH. Although several case control studies have reported an association between *CYP1A1 *polymorphism and susceptibility to a number of adult cancers, others reported no evidence of such association [19-22].

Glutathione S-transferases (GSTs) are a family of enzymes that carry out a wide range of functions in cells. GSTs also play a role in detoxification of a variety of endogenous and exogenous electrophilic compounds, such as the removal of reactive oxygen species and regeneration of S-thiolated proteins that are products of oxidative stress and the detoxification of carcinogenic compounds. In addition to this, GSTs modulate the induction of other enzymes and proteins important for cellular functions, such as DNA repair [23]. This class of enzymes is therefore important for maintaining cellular genomic integrity and as a result may play an important role in cancer susceptibility. Several studies have shown that *GSTM1 *0/0 is associated with an increased risk of lung, bladder, gastric, colorectal, and laryngeal cancers [24-28], although not of skin cancer development [29]. Thus carcinogenesis could be related to the absence of the functional GSTM1 alleles. In the thyroid, many studies related to genes such as *p53, RAS RET *and thyrotropin receptor have improved our understanding of thyroid carcinogenesis [30].

We investigated the association between polymorphisms in genes encoding folic acid metabolizing enzymes MTHFR and six xenobiotics-metabolizing enzymes including *CYP1A1 T3801C, C4887A, GSTP1 A1578G, GSTP1 C2293T, GSTM1, GSTT1*, NAT2 G590A, NQO*1 C609T, since genetic differences in these genes are highly race specific and have never been screened in the Saudi PTC cases. We hypothesized that polymorphisms of genes responsible for drug metabolism xenobiotic genes may be associated with risk of thyroid cancer.

## Methods

### Study Subjects

Formalin fixed, paraffin embedded samples from 223 newly presenting and previously untreated Arabian patients with papillary thyroid carcinoma (PTC) were investigated. PTC patient samples (n = 223) were readily available from the archives of the Pathology Department at King Faisal Specialist Hospital and Research Centre (KFSHRC). The Institutional Review Board of the King Faisal Specialist Hospital and Research Centre approved the study in accordance with the Declaration of Helsinki. Diagnosis was confirmed by pathologic review using the diagnostic criteria defined in the WHO Classification [31]. Briefly, tissue cylinders with a diameter of 0.6 mm were punched from representative tumor regions of each "donor" tissue block by using a home made semiautomatic robotic precision instrument as described [32]. Genomic DNA was extracted from paraffin embedded PTC tissues using the Puregene DNA isolation kit (Gentra, Minneapolis, MN) following the manufacturer's recommendations. Genotypes frequency data of *GSTM1, GSTT1, CYP1A1 *T3801C and C4887A, *MTHFR *C677T, *GSTP1 *A1578G and C2293T, NQO*1 C609T and *NAT2 *G590A for control population utilized in this report was from our previous study [33], briefly, whereby peripheral blood was obtained from age matched, 513 individual healthy blood donors of Middle Eastern Arab origin (95% Saudi Arabians and 5% from other Arab countries such as Jordan, Syria, Lebanon, Yemen) visiting the Blood Bank at KFSHRC, Riyadh, Saudi Arabia.

### Genotyping

We first determined that the use of Hotstart Taq polymerase allowed us to apply the same PCR conditions to optimally amplify each of the variant alleles for all genes studied. PCR reactions were performed in 25 μl containing 50 ng of genomic DNA, 12.5 pmol each primer, 200 μM of each dNTPs, 2.5 mM MgCl_2 _and 0.5 units Hotstart Taq DNA Polymerase. After denaturation for 10 minutes at 95°C, each PCR was performed for 35 cycles of 1 minute at 95°C, 1 minute at 59°C and 1.5 minutes at 72°C. The final elongation step was 10 minutes. Negative non-template controls were included in each PCR. The same aliquot of DNA was used to analyze all SNPs. PCR products were directly analyzed on a 2.5% agarose gel.

#### MTHFR Polymorphisms

*MTHFR C677T *was amplified using primers: (5-TGAAGGAGAAGGTGTCTGCGGGA-3 and 5-AGGACGGTGCGGTGAGAGTG-3), the *MTHFR C677T *genotypes CC (wildtype) produced one 198-bp band, *MTHFR *TT (homozygote mutation) produced two bands of 175-bp and 23-bp, while *MTHFR C677T *CT (heterozygote) samples exhibited a digestion pattern of all three bands when digested with Hinf I.

#### CYP1A1 Polymorphisms

Using primers: (5-GGCTGAGCAATCTGACCCTA-3 and 5-TAGGAGTCTTGTCTCATGCCT-3), the *CYP1A1 T3801C *genotypes TT (wildtype) produced a 341-bp band, CYP1A1 CC (homozygote mutation) produced two bands of 175-bp and 166-bp, while *CYP1A1 T3801C *TC (heterozygote) samples exhibited a digestion pattern of all three bands when digested with MSP I. *CYP1A1 C4887A *gene (rs1048943) was determined using primers (5-CTGTCTCCCTCTGGTTACAGGAAGC-3 and 5-TTCCACCCGTTGCAGCAGGATAGCC-3), and Bsa I was used to digest the 204-bp product: *CYP1A1 C4887A *CC (wildtype) produced two bands, a 138-bp and 66-bp, *CYP1A1 C4887A *AA (homozygote mutation) produced one band of 204-bp, while *CYP1A1 C4887A *CA (heterozygote) samples exhibited a digestion pattern of all three bands (204-bp, 138-bp and 66-bp) when digested with Bsa I.

#### GSTP1 Polymorphisms

Using primers: (5-GGCTCTATGGGAAGGACCAGCAGG-3 and 5-GCACCTCCATCCAGAAACTGGCG-3), the *GSTP1 A1578G *(rs947894) genotype AA (wildtype) produced a 256-bp band, *GSTP1 *GG (homozygote mutation) produced two bands of 204-bp and 52-bp, while *GSTP1 A1578G *AG (heterozygote) samples exhibited a digestion pattern of all three bands when digested with BsmA I. Similarly, using primers: (5-CAGCAGAGGCAGCGTGTGTGC-3 and 5-CCCACAATGAAGGTCTTGCCTCC-3), the *GSTP1 C2293T *(rs1799811) CC (wildtype) produced two bands, a 120-bp and 97-bp, *GSTP1 C2293T *TT (homozygote mutation) produced one band of 217-bp, while *GSTP1 C2293T *CT (heterozygote) samples exhibited a digestion pattern of all three bands (217-bp, 120-bp and 97-bp) when digested with Aci I.

#### NQO*1 Polymorphisms

NQO*1 C609T was amplified using primers: (5-AGTGGCATTCTGCATTTCTGTG-3 and 5-GATGGACTTGCCCAAGTGATG-3), the NQO*1 C609T genotypes CC (wildtype) produced two bands, a 188-bp and 85-bp band, NQO*1 TT (homozygote mutation) produced three bands of 151-bp, 85-bp and 37-bp, while NQO*1 C609T CT (heterozygote) samples exhibited a digestion pattern of all four bands when digested with Hinf I.

#### NAT2 Polymorphisms

*NAT2 G590A *was amplified using primers: (5-CCTGGACCAAATCAGGAGAG-3 and 5-ACACAAGGGTTTATTTTGTTCC-3), the *NAT2 G590A *genotypes GG (wildtype) produced three bands, a 170-bp, 139-bp and 112-bp band, *NAT2 *AA (homozygote mutation) produced two bands of 309-bp and 112-bp, while *NAT2 G590A *GA (heterozygote) samples exhibited a digestion pattern of all four bands when digested with Taq I.

#### GSTM1 and GSTT1 Polymorphisms

The presence or absence of *GSTM1 *and *GSTT1 *was determined by multiplex PCR in which β-globin gene was co-amplified as an internal control. *GSTM1 *was amplified using primers 5-GAACTCCCTGAAAAGCTAAAGC-3 and 5-GTTGGGCTCAAATATACGGTGG-3. Wherever present *GSTM1 *produced a 219-bp band along with 110-bp *β-globin *amplified with primers 5-ACACAACTGTGTTCACTAGC-3 and 5-CAACTTCATCCACGTTCACC-3. Similarly, *GSTT1 *produced 480-bp band along with 110 *β-globin *band. *GSTT1 *was amplified using primers 5-TTCCTTACTGGTCCTCACATCTC-3 and 5-TCACCGGATCATGGCCAGCA-3. Absence of 219-bp band for GSTM1 or 480-bp for *GSTT1 *with the presence of 110 *β-globin *was recorded as deleted.

### Statistical Analysis

Univariate analysis using Chi-square analyses (two-sided) was first performed to compare the differences in genotype and allele frequencies between case and control subjects. The odds ratio (OR) and 95% confidence interval provide a measure for the strength of association, e.g. indicating the increase in the odds of a given benign or malignant thyroid tumor demonstrating a particular genotype compared with the control population. Statistical significance of the differences in the frequency of genotypes was assessed, applying two-sided Fisher's exact test, and odds ratios (ORs) with 95% CI were calculated on the SPSS statistical package (version 11.0). All tests were conducted at the p < 0.05 level of significance.

## Results

Tables 1, 2 provide detailed information on the frequency of the multiple genotypes analyzed. 223 PTC samples were included in this study. Initially, only 50 out of 223 PTC samples were analyzed for 9 SNPs in 7 genes (Table 1). Furthermore, comparing cases with the control study group the association between polymorphisms and the risk to develop thyroid cancer was initially assessed with 50 PTC samples and the SNP's that showed statistical significance were selected to further confirm the finding with larger number of cases analyzed (n = 223) (Table 2).

**Table 1:** Distribution of polymorphisms in healthy population and thyroid cancer patients (n = 50).

**Polymorphism**	**Genotype **	**Control**	**PTC**	p*	**OR^$^**	**95% CI**
*MTHFR C667T*	CC	372(72.8%)	30(61.2%)			
	CT	126(24.7%)	18(46.15%)	0.09	1.77	0.96–3.29
	TT	13(2.5%)	1(2.6%)	1	0.95	0.12–7.54
	CT+TT	139(27.2%)	19(48.7%)	0.10	1.70	0.92–3.11
						
*CYP1A1 T3801T*	TT	327(64.2%)	27(57.4%)			
	TC	157(30.9%)	15(31.9%)	0.73	1.16	0.60–2.24
	CC	25(4.9%)	5(10.6%)	0.09	2.42	0.86–6.83
	CC+TC	182(35.8%)	20(42.5%)	0.35	1..33	0.73–2.44
						
*CYP1A1 C4887A*	CC	331(64.8%)	7(16.7%)			
	CA	162(31.7%)	30(71.4%)	< 0.0001	8.76	3.77–20.36
	AA	18(3.5%)	5(11.9%)	< 0.0001	13.14	3.79–45.47
	CA+AA	180(35.2%)	35(83.3%)	< 0.0001	9.19	4.00–21.12
						
*GSTP1 A1578G*	AA	170(33.5%)	18(38.3%)			
	AG	271(53.5%)	25(53.2%)	0.74	0.87	0.46–1.65
	GG	66(13%)	4(8.5%)	0.45	0.57	0.19–1.75
	AG+GG	337(66.5%)	29(61.7%)	0.52	0.81	0.44–1.51
						
*GSTP1 C2293T*	CC	389(76.3%)	30(75%)			
	CT	113(22.2%)	8(20%)	1	0.92	0.41–2.06
	TT	8(1.5%)	2(5%)	0.17	3.24	0.66–15.95
	CT+TT	121(23.7%)	10(25%)	0.85	1.07	0.51–2.26
						
*GSTM1*	P	233(45.4%)	24(48%)			
	D	280(54.6%)	26(52%)	0.77	0.90	0.50–1.61
						
*GSTT1*	P	385(75%)	16(32.7%)			
	D	128(25%)	33(67.3%)	< 0.0001	6.24	3.31–11.64
						
*NAT2 G590A*	GG	284(55.4%)	19(46.3%)			
	GA	181(35.3%)	17(41.5%)	0.34	1.40	0.71–2.77
	AA	47(9.2%)	5(12.2%)	0.37	1.59	0.57–4.47
	GA+AA	228(44.5%)	22(53.7%)	3.29	1.44	0.76–2.73
						
NQO*1 C609T	CC	295(58.5%)	30(61.2%)			
	CT	177(35.1%)	18(36.7%)	1	1	0.54–1.85
	TT	32(6.4%)	1(2.04%)	0.34	0.31	0.04–2.33
	CT+TT	209(41.5%)	19(38.74%)	0.76	0.89	0.49–1.63

* Statistical significance of the differences in the frequency of genotypes was assessed, applying two-sided Fisher's exact test to compare between case and control subjects.

^$ ^The odds ratio (OR) providing a measure for the strength of association demonstrating a particular genotype compared with the control population calculated using the SPSS statistical package (version 11.0).

P = Present and D = Deletion for *GSTM1 and GSTT1 *gene

**Table 2:** Distribution of Xenobiotic genes polymorphisms in healthy population and PTC cases (n = 223).

**Polymorphism**	**Genotype**	**Control group**	**PTC Cases**	p*	**OR^$^**	**(95%CI)**
*CYP1A1 C4887A*						
	CC	331 (64.8%)	95 (47%)			
	CA	162 (31.7%)	89 (44%)	< 0.0001	1.91	(1.36–2.70)
	AA	18 (3.5%)	18 (10%)	< 0.001	3.48	(1.74–6.96)
	CA+AA	180 (35.2%)	107 (53%)	< 0.0001	2.07	(1.49–2.88)
						
*NAT2 G590A*						
	GG	284 (55.4%)	99 (50%)			
	GA	181 (35.3%)	81 (41%)	0.180	1.28	(0.91–1.82)
	AA	47 (9.2%)	18 (9%)	0.76	1.11	(0.61–1.98)
	GA+AA	228 (44.5%)	99 (50%)	0.21	1.25	(0.90–1.73)
						
*GSTT1*						
	P	385 (75%)	96 (46.37%)			
	D	128 (25%)	111 (53.62%)	< 0.0001	3.48	(2.48–4.88)
						
*GSTM1*						
	P	233 (45.4%)	109 (53.7%)			
	D	280 (54.6%)	94 (46.3%)	0.047	0.72	(0.52–0.99)
						
*GSTM1+GSTT1*						
	P	423 (82.8)	159 (78.32%)			
	D	88 (17.2%)	48 (23.64%)	0.074	1.45	(0.98–2.16)

* Statistical significance of the differences in the frequency of genotypes was assessed, applying two-sided Fisher's exact test to compare between case and control subjects.

^$ ^The odds ratio (OR) providing a measure for the strength of association demonstrating a particular genotype compared with the control population calculated using the SPSS statistical package (version 11.0).

P = Present and D = Deletion for *GSTM1 and GSTT1 *gene

Frequencies for *CYP1A1 *C4887A genotypes CC, CA, AA and CA+AA were 16.7%, 71.4%, 11.9% and 83.3% respectively and frequency of A allele was 48% when initial analysis with 50 cases was performed. Genotype CA showed 8.7-fold, AA showed 13.1-fold and CA+AA demonstrated 9.2-fold higher risk, compared with wild type (p < 0.0001). On the other hand, analysis of *CYP1A1 *T3801C showed no statistical significance (Table 1).

Although *GSTP1 *C2293T, genotype TT accounted for 5% of PTC cases comparing with 1.5% of controls and showed 3.24 fold higher risk to develop thyroid cancer but did not reached to level of significance (Table 1).

Among 50 PTC cases, 67.3% were homogenous for the *GSTT1 *null genotype compared with 25% of controls. Consequently there was 6.24 fold increase in the risk of thyroid cancer associated with *GSTT1 *null genotype (OR = 6.24). No significant differences were found in the frequency of other genes when 50 cases were compared with controls (Table 1).

Using our selection criteria, in particular two genes from xenobiotic metabolizing enzyme system (*CYP1A1 C4887A *and *GSTT1*) were chosen to elaborate the study with further 173 cases, since they have demonstrated high statistical significance and odds ratio when controls were compared to initially analyzed 50 cases form PTC samples. Additionally two genes, *GSTM1 *and *NAT2 G590A*, which have shown no statistical significance, were also selected for further analysis to confirm the reliability of our selection criteria. 173 further PTC cases were analyzed for these four genes and compared with control case group to assess the association between polymorphisms and the risk to develop thyroid cancer. Although the initial selection criteria was not deemed statistically powerful due to less number of samples nevertheless provided with the selection of genes to expand the study with statistically significant data. As can be seen *GSTM1 *and *NAT2 G590A *genes, demonstrated no significant association with risk estimate compared to control group even when expanded with 173 more PTC samples (tables 1 and 2).

Among selected genes, frequencies for *CYP1A1 C4887A *genotypes CC, CA, AA and CA+AA were 47%, 44%, 9% and 53% respectively and frequency of A allele was 31%. Genotypes CA, AA and variant allele A demonstrated significant differences comparing to wild type genotype CC. Odds ratio showed CA has 1.9 fold, while AA has 3.4 fold and variant allele A showed 2 fold greater risk of developing thyroid cancer comparing to wild type.

*GSTT1 *null showed 3.48 times higher risk of developing thyroid cancer (p < 0.0001) while *GSTM1 *null showed protective effect (p < 0.05, OR = 0.718). Although the *GSTT1 *null was risk factor (OR = 3.48) to develop PTC (p < 0.0001) but double null of *GSTT1 *and *GSTM1 *showed no statistical significance (Table 2).

We also investigated whether the prevalence of combined *GSTT1 *null and *GSTM1 *null genotype (double-null genotype) was significantly increased in PTC cases compared with controls (Table 2). Among PTC cases, 23.6% were both *GSTT1 *and *GSTM1 *null, compared with 17.2% of controls. When individuals with both *GSTT1 *and *GSTM1 *were considered as the reference group, analysis demonstrated comparatively lower increased risk (OR = 1.45) in individuals with the double-null genotype than that seen with *GSTT1 *null alone, but the results did not reach the level of significance.

## Discussion

Multiple enzyme pathways are involved in detoxification of chemotherapeutic agents and/or carcinogens. Variations in *GSTT1*, *GSTM1 *and *GSTP1 *have been previously demonstrated to influence drug efficacy and toxicity and also to modify individual susceptibility to cancers [34-39] however, there are scarce data specific to patients with thyroid cancer [40-46]. Host factors may contribute to an individual's risk of developing secondary cancers. Our findings suggest that polymorphisms of certain xenobiotic metabolizing enzyme genes modify the individual susceptibility to develop thyroid cancer in the Saudi population. Our findings from this hospital-based case-control study are not entirely consistent with previous published studies.

GSTs participate in the metabolism of alkylating agents, anthracyclines and steroids and variations in within these genes can significantly influence treatment outcome [35,36]. The different glutathione S-transferase (GST) enzymes have classically been considered as an important part of the cell defense against numerous harmful chemicals and reactive oxygen species (ROS) produced endogenously and in the environment [48-52]. Their importance is suggested by the finding that mutations in *GST *genes have been associated with susceptibility to various diseases, in particular with cancer [48].

The only three studies published so far on involvement of *GSTT1 *and *GSTM1 *null alleles in thyroid cancer risk were carried out in three different regions with different frequencies of *GST *deletion genotype in general population (Spain, Brazil and Portugal) [45,52,53]. Contrary to our findings the results obtained in three recently published studies [45,52,53] did not show increased risk between the *GST *polymorphisms and papillary and follicular thyroid cancer susceptibility. We found that the *GSTT1 *null genotype was associated with high risk (OR = 3.478, p < 0.0001) of developing PTC. Although our findings, showed association between the *GSTM1 *null genotype with decreased risk against development of PTC (OR = 0.718, p < 0.05) comparing to control group, is consistent with previous reports with other cancers [19,20,52,53] including thyroid cancer [43]. Contrary to our findings Canbay *et al*., in a short study with 32 thyroid samples and 44 controls demonstrated association of *GSTM1 *null genotype with high risk of developing thyroid cancer in patients stratified with age [46]. Other thyroid cancer studies showed that only the combination of *GSTM1 *and *GSTT1 *null inheritance [53] or only the presence of three potentially risk alleles, namely *GSTM1*-null, *GSTT1*-null and *GSTP1 A1578G *genotype GG [46], leads to significant increase in adjusted odds ratio for papillary and follicular thyroid cancers. These results were not confirmed by the third study conducted on a Spanish population [52]. Our data showed weak association with double null genotypes *GSTT1*+*GSTM1*, and risk of PTC (OR = 1.451, p = 0.074) (Table 2). Although with limited number of cases (n = 50) we did not find any statistical association with *GSTP1 A1578G *genotypes but nonetheless our findings in PTC for *GSTP1 C2293T *with genotypes TT showed non-significant association with elevated risk of PTC (OR = 3.242).

The *GSTT1 *and *GSTM1 *null allele frequency among controls in the Brazilian, Spanish and other European studies [52] demonstrated marked variation compared to ours (17.2% vs. 5, 10.3 and 9.2%). Thus, the differences in our finding with published data could indicate role of these genotypes distinct to Saudi population. Morari *et al*. [53] consistent with our data showed that papillary carcinoma patients (17%) and follicular carcinoma patients (10%) presented a higher prevalence of the combined *GSTT1/M1 *null genotype than the normal population individuals (5%; OR 2.6; 95% CI, 1.044–6.355; p < 0.05). Similar to others [52,53] our results demonstrated higher frequency of combined *GSTT1/M1 *null genotype in PTC than in control group 23.6% vs. 17.2%). In addition, the frequency of null *GSTT1 *in our patients was two fold higher than in control group (53.6% vs. 25%).

In the present study, an increased risk of developing PTC associated with the *GSTT1 *null and protective effect of *GSTM1 *null allele was suggested, although not reaching the statistical significance but demonstrated trend towards increased risk with double, *GSTT1 *and *GSTM1 *null genotype.

*CYP1A1 *genes are associated with higher enzymatic inducibility. Levels of circulating hormone are affected by genetic variability, and analysis of genetic polymorphisms that influence estrogen production may provide insight into its potential role in carcinogenesis. Estrogen exerts pleotropic effects on lymphocyte activation, proliferation, cell cycle progression and apoptosis [56], factors that may influence thyroid cancer risk. Additionally depending on levels, cortisol can either suppress or stimulate immune function, so modulation of its production could potentially influence thyroid cancer risk. In our study, *CYP1A1*, phenotype AA demonstrated strong association with increased risk of developing PTC (OR = 3.484, p < 0.001). Additionally other *CYP1A1 C4887A *genotypes CA (OR = 1.914, p < 0.0001) and variant allele A (OR = 2.071, p < 0.0001) also demonstrated two fold high risk of developing PTC compared to the wild type. Recently we also demonstrated significantly high differential expression of *CYP1A1 *gene in PTC compared to normal counterparts, support the notion and may suggest a possible role for estrogen and possibly other cholesterol metabolites formed via *CYP1A1 C4887A *in the pathogenesis of PTC [57]. Scarce data is available on the association between *CYP1A1 *polymorphism and increased susceptibility to thyroid cancer. Recently Bufalo *et al*., in an extensive study investigating *CYP1A1 (m1 and m2) *role in thyroid tumorigenesis and its connection with *GSTM1*, *GSTT1*, *GSTP1*, *GSTO1*, and codon 72 of *p53 *genotypes, demonstrated an inverse association between germline *CYP1A1 *inheritance and smoking with the risk of thyroid nodules and papillary carcinomas [58].

The reasons for these divergent results are not clear but are likely racial differences because the frequencies of *CYP1A1*, *GSTT1*, *GSTM1*, *GSTP1 *and *NQO1 *in our controls were different from those found in other studies [33]. For instance certain SNPs like *CYP1A1 *A4889G and *GSTP1 *A1578G were not in Hardy-Weinberg equilibrium in the control samples which is contradicting comparing to other population based data. In order to question the specificity of the assay and to rule out the possibility of uncompleted digestion due to inactivation of enzymes or inhibitors in digestion mixture, we repeated the assays multiple and over period of time. Consistent results were obtained demonstrating this to be the true feature in our population.

Consideration must also be given to potential limitation of the present study and that is the nature of the control subjects enrolled which weakens the study to be claimed purely case: control. Although controls were matched with respect to age but there was gender differences, with preponderance of male blood donors (25:1). While this initial documentation of allelic frequencies in the Arabs may be biased because of the nature of subjects enrolled (preponderance of male blood donors), previous reports have indicated only minimal influence of age and gender on the distribution of these polymorphisms in a given population.

Additionally, in our study PTC patient samples from paraffin embedded tissues were readily available from the archives of the Pathology Department at King Faisal Specialist Hospital and Research Centre (KFSHRC). Similarly control group was randomly selected from DNA available from peripheral blood samples at institutional Tissue Biorepository Bank at KFSHRC. It would have been ideal to analyze samples from uniform source (tissue only or peripheral blood of patients and controls) but it was not possible in our case since archival samples were used. Since genetic aberrations, that could be present in the tumor but possibly not in the "normal tissue" could confound analyses but to the best of our knowledge there are no such reports suggesting somatic mutations in studied genes in thyroid cases.

Obviously, further study with a larger sample size is needed to confirm these findings and to examine the interaction between genotypes. However, this study does provide preliminary case-control data on several polymorphisms in genes of the xenobiotic pathways and risk to develop thyroid cancer.

## Conclusion

We hypothesized that polymorphisms of genes responsible for drug metabolism xenobiotic genes may be associated with risk of thyroid cancer. Of the 9 polymorphisms screened, we identified *GST, GSTM1 and CYP1A1 C4887A *may be of importance to this disease process. Obviously, confirmatory studies with larger sample sizes will be needed for other genes that showed no significance with risk, but these findings represent a step toward the understanding of genetic susceptibility to thyroid cancer. If these findings are confirmed, then genotypes could be incorporated into future genetic profiles of thyroid cancer risk and may serve in future cancer prevention efforts or public health responses to accompanying risk factors.

## Competing interests

The authors declare that they have no competing interests.

## Authors' contributions

AKS participated in the data analysis and drafting of the manuscript; MI and MAR took part in the genotyping, managed and analyzed the data and approved the final version of the manuscript; JA and RB participated in analyzing subset of patient samples by sequencing, validating RFLP and approved the final version of the manuscript; SUS participated in collecting the clinical data analysis and approved the final version of the manuscript; FAD, OAS and AAN contributed substantially in acquisition of samples and interpretation of data and also participated in revising and formulating the content. SU participated in the concept and design of the study and approved the final version of the manuscript; KAK was responsible for the conception, funding, design and coordination of the study.

## Pre-publication history

The pre-publication history for this paper can be accessed here:


